# Wild-Type Domestication: Loss of Intrinsic Metabolic Traits Concealed by Culture in Rich Media

**DOI:** 10.1007/s00248-024-02459-z

**Published:** 2024-11-21

**Authors:** Ben Vezina, Helena B. Cooper, Jessica A. Wisniewski, Matthew H. Parker, Adam W. J. Jenney, Kathryn E. Holt, Kelly L. Wyres

**Affiliations:** 1 Department of Infectious Diseases, School of Translational Medicine, Monash University, Melbourne, VIC Australia; 2 Centre to Impact AMR, Monash University, Clayton, VIC Australia; 3 Alfred Pathology Service, Microbiology Unit, The Alfred Hospital, Melbourne, VIC Australia; 4 Department of Infection Biology, London School of Hygiene and Tropical Medicine, London, UK

**Keywords:** Metabolism, Klebsiella pneumoniae, Attenuation, Whole genome sequencing, Nutrient auxotrophy

## Abstract

Bacteria are typically isolated on rich media to maximise isolation success, removing them from their native evolutionary context. This eliminates selection pressures, enabling otherwise deleterious genomic events to accumulate. Here, we present a cautionary tale of these ‘quiet mutations’ which can persist unnoticed in bacterial culture lines. We used a combination of microbiological culture (standard and minimal media conditions), whole genome sequencing and metabolic modelling to investigate putative *Klebsiella pneumoniae* L-histidine auxotrophs. Additionally, we used genome-scale metabolic modelling to predict auxotrophies among completed public genomes (*n* = 2637). Two sub-populations were identified within a *K. pneumoniae* frozen stock, differing in their ability to grow in the absence of L-histidine. These sub-populations were the same ‘strain’, separated by eight single nucleotide variants and an insertion sequence-mediated deletion of the L-histidine biosynthetic operon. The His^−^ sub-population remained undetected for > 10 years despite its inclusion in independent laboratory experiments. Genome-scale metabolic models predicted 0.8% public genomes contained ≥ 1 auxotrophy, with purine/pyrimidine biosynthesis and amino acid metabolism most frequently implicated. We provide a definitive example of the role of standard rich media culture conditions in obscuring biologically relevant mutations (i.e. nutrient auxotrophies) and estimate the prevalence of such auxotrophies using public genome collections. While the prevalence is low, it is not insignificant given the thousands of *K. pneumoniae* that are isolated for global surveillance and research studies each year. Our data serve as a pertinent reminder that rich-media culturing can cause unnoticed wild-type domestication.

## Introduction

Microbial species often display high levels of genetic diversity [1, 2], which can drive ecological and pathogenic differentiation. Whole genome sequencing of environmental and clinical microbes is crucial for understanding microbial diversity and distribution [3, 4], predicting antimicrobial resistance and virulence [5]. Prior to sequencing, isolates are enriched using nutrient-dense growth media like Luria–Bertani (LB) to maximise isolation success in large-scale studies [3, 4]. However, what often goes unacknowledged is that isolation and sequencing represent a snapshot of an organism. Genomes and populations are in constant flux, guided by evolutionary selection pressures such as competition for space [6] and nutrients [7], bacteriophage predation [8] and antibiotics [9]. Growth in rich media removes these pressures, permitting deleterious genomic events to accumulate. These ‘quiet mutations’ may go unnoticed or incorrectly accepted as wild-type characteristics. In this study, we present a cautionary tale of quiet mutations, how they can be present in primary frozen stocks and persist unnoticed. We also estimate the frequency of these events across public *Klebsiella pneumoniae* Species Complex (*Kp*SC) genomes.

## Methods

### Bacterial Strains and Culture

**Fig. 1:**
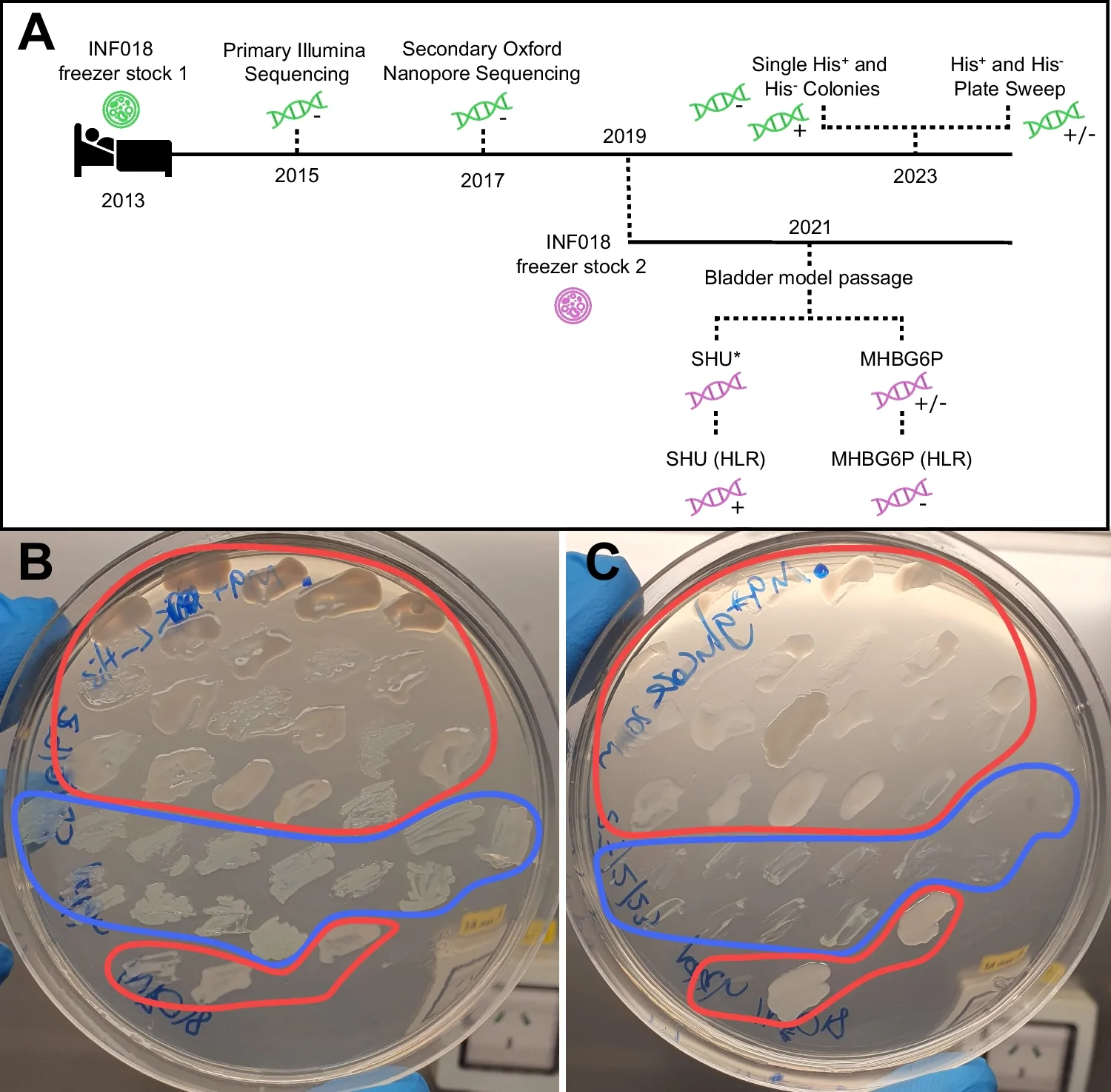
**a** Timeline of *K. pneumoniae* INF018, with dark lines representing different frozen stocks (green and purple) and dotted lines showing independent subculturing events. Sequencing is represented with a DNA icon coloured by the frozen stock they originated from, with a “ + ” indicating a His^+^ colony, “ − ” indicating a His^−^ colony and “ + / − ” indicating mixed colonies. Sequencing for the in vitro bladder model was done using INF018 passaged in either cation-adjusted Mueller–Hinton broth (MHBG6P) or synthetic human urine (SHU), including high-level Fosfomycin-resistant (HLR) subpopulations from each media [12]. Sequencing data for INF018 SHU (labelled with an asterisk) is unavailable as the genome failed quality control [12]. **B and C** Patch plates showing heterogeneous population of His^+^ and His^−^ sub-populations within the 2013 frozen stocks. Colonies within the red boundaries show *K. pneumoniae* INF018, whereas the blue boundary shows *K. variicola* subsp. *variicola* INF232. Colonies match positions on each plate. **B** M9 agar + 20 mM L-histidine. **C** M9 agar + 20 mM D-glucose

Two *Kp*SC isolates collected from hospitalised patients in Melbourne, Australia were used in this study: *K. pneumoniae* INF018 and *K. variicola subsp. variicola* INF232, both causing urinary tract infections [5]. INF018 was part of a reported transmission cluster [10] and was used in two subsequent studies focusing on metabolism [5, 11] and one exploring passage through an in vitro bladder model [12], from which additional genome sequences were generated (Fig. 1A). Isolates were grown as previously reported [13]. Briefly, 5 mL cultures were grown at 37 °C, shaking at 200 RPM overnight in broth: M9 minimal media (Sigma) containing either 20 mM D-glucose, 20 mM L-histidine or both. Isolates were plated onto LB or M9 plates containing the same substrates.

### DNA Isolation and Sequencing

Overnight cultures were pelleted at 8000 × g and DNA extracted using GenFind V3 (Beckman Coulter). DNA libraries were constructed using Illumina DNA Library Prep Kit, (M) Tagmentaton (Illumina) and sequenced on the Illumina NovaSeq 6000 at 2 × 250 bp. Reads and assemblies were deposited at Genbank (BioProject PRJNA1115910, further details on Figshare 10.6084/m9.figshare.25864864).

### Genomic Analysis

Genomes were assembled using Unicycler v0.4.7 with the –keep 0 option [14] and annotated with Bakta v1.8.1 [15] using the ‘–gram – ‘ option and v1.8.1 database. Kaptive 3 [16, 17] was used for determining K/O locus intactness. Read and contig mapping to a completed genome representing a His^+^ isolate from the same transmission cluster (accession: GCA_904863215.1) was performed with minimap2 v2.26 [18], using SAMtools v1.19 [19] for depth estimation. Genome-wide single nucleotide variant (SNV) distances were calculated via read mapping and variant calling against the completed INF018 genome (accession: SAMEA3356961, His^−^) using RedDog v1beta.11 (https://github.com/katholt/RedDog). ISEScan v1.7.2.3 [20] was used to identify Insertion Sequences.

Completed public *Klebsiella* genomes (*n* = 2637) were obtained from Genbank, accessed: 29/01/2024 (10.6084/m9.figshare.25864864). Bactabolize [21] v1.0.2 was used to construct metabolic models (using the *Kp*SC pan v2.0.2 reference) and predict growth phenotypes [13].

## Results

### Heterogeneous Sub-Population in Clinical Isolate Sample

We previously reported metabolic models [13] which unexpectedly predicted L-histidine auxotrophies for two isolates (*K. pneumoniae* INF018 and *K. variicola* INF232). L-histidine biosynthesis is essential for cellular growth and protein production and is conserved across all bacteria [22]. Auxotrophic strains are unable to produce L-histidine and therefore cannot grow in environments lacking this substrate. Both genomes were complete [5], ruling out misassembly as an explanation, and both isolates were subsequently tested in vitro to confirm L-histidine auxotrophy [13]. In parallel, another team member, unaware of these data, performed an independent experiment using the same 2013 INF018 frozen stock, which demonstrated growth in M9 + D-glucose without L-histidine.

When these inconsistencies were realised, the 2013 frozen stocks were plated onto LB and colonies patched onto M9 + D-glucose and M9 + L-histidine (Fig. 1B, C). All colonies grew on L-histidine, 19/23 INF018 colonies grew on D-glucose without L-histidine, while 0/13 INF232 colonies were able to grow on D-glucose without L-histidine. This confirmed the 2013 INF018 frozen stock was a heterogeneous population, consisting of His^+^ and His^−^ sub-populations, while INF232 was entirely His^−^. Compared to His^+^, His^−^ mutants displayed a flatter, paler colony morphology. It was unclear if these morphological differences were driven by the direct impact of the auxotrophy, or by independent genetic changes.

### Genomics Confirmed Deletion of L-Histidine Biosynthetic Operon

Single INF018 His^+^ and His^−^ colonies from the 2013 frozen stock were whole-genome sequenced to confirm the heterogeneous population (Fig. 1). Compared to the complete INF018 genome [5], the INF018 His^+^ genome had eight SNVs, while INF018 His^−^ had zero, indicating they were the same strain [23]. Gorrie et al. had previously reported the INF018 frozen stock contained an ESBL plasmid (*bla*_CTX-M-15_), which was lost during culture and original sequencing [5], but we recovered the plasmid in both His^+^ and His^−^ colony genomes.

**Fig. 2:**
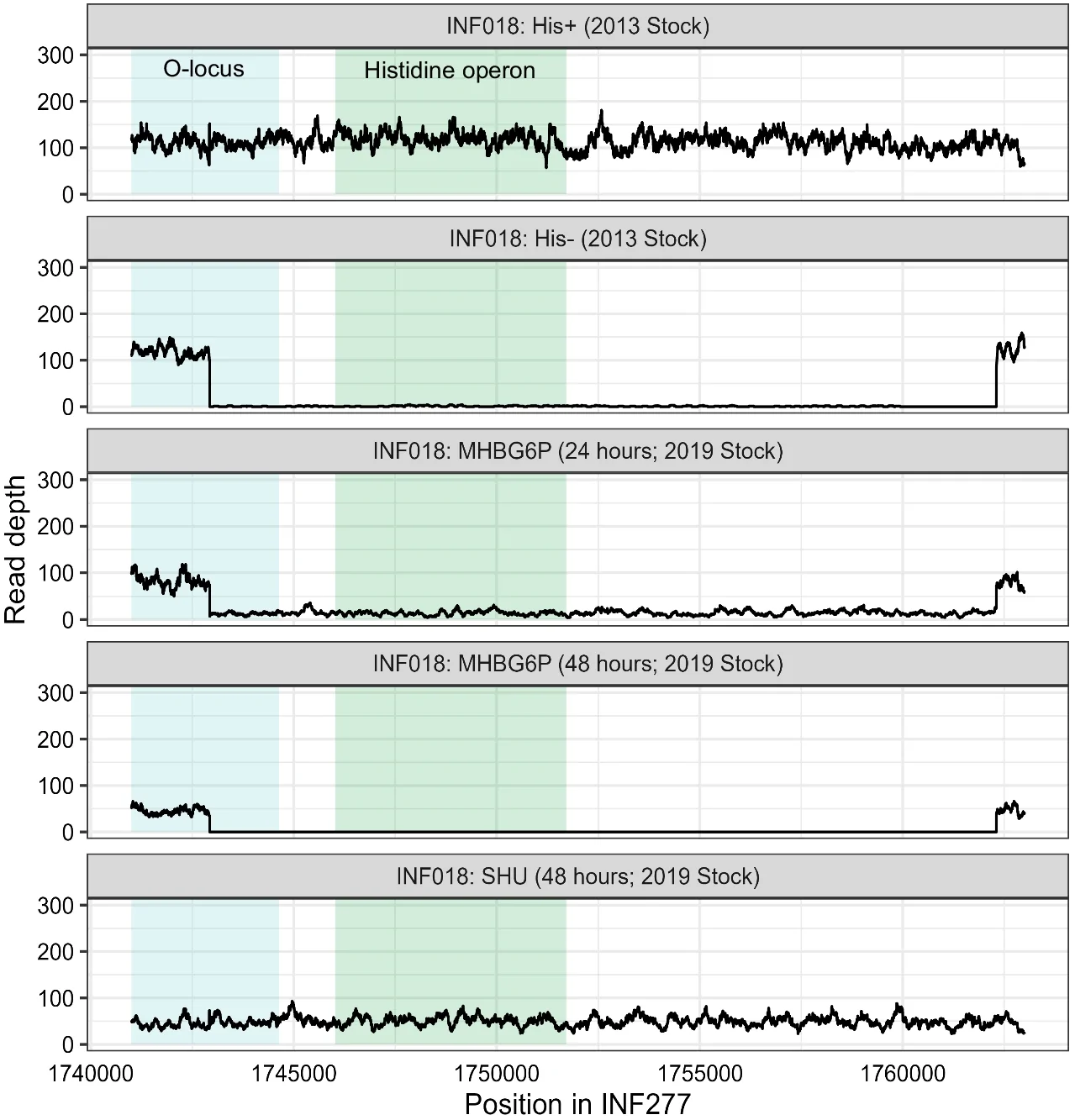
Read mapping of experimentally verified INF018 His^+^ and His^−^ strains (2013 frozen stock) as well as INF018 reads from the in vitro bladder model experiment (accessions: SAMN17846711, SAMN17846713 and SAMN21465650) [12] wherein INF018 (2019 frozen stock) was passaged in cation-adjusted Mueller–Hinton broth (MHBG6P) or synthetic human urine (SHU), prior to sequencing at 24 and 48-h time-points. High-level Fosfomycin-resistant (HLR) subpopulations from each passage were also sequenced [12]. Reads were aligned to *K. pneumoniae* INF277 (accession: SAMN06112222, His +), which is part of the same transmission cluster as INF018 [5]. The green box highlights the location of the L-histidine biosynthesis operon, and the blue box highlights the 3′ region of the O-antigen locus. Mean sequencing depth for 2013 INF018 genomes: His^+^ (108.9x), His.^−^ (113.88x); 2019 INF018 genomes: MHBG6P 24 h (82.11x), MHBG6P 48 h (49.48x), SHU 48 h (52.27x)

Read mapping indicated the INF018 His^−^ sub-population had a 19,388 bp deletion spanning the L-histidine biosynthetic operon (*his*) and part of the adjacent O antigen biosynthetic locus (Fig. 2). The chromosomal deletion event was likely mediated by insertion of IS5_222 introduced into the genome on the ESBL plasmid, and found exclusively on plasmids in closely related His^+^ *K. pneumoniae* strains [5]. The loss of O-antigen would prevent K-antigen anchoring, which may have contributed to the colony morphology variation (Fig. 1B, C). Notably, the K and O antigens are also major *Klebsiella* pathogenicity determinants and targets for novel anti-*Klebsiella* vaccines [24]. Antigen variant prioritisation is informed by genomic analyses which would have predicted a misleading O-antigen deficiency in this case.

Prior to this work, INF018 was used in an in vitro bladder model experiment [12] where it was passaged in cation-adjusted Mueller–Hinton broth (MHBG6P) or synthetic human urine (SHU) with/without fosfomycin and subjected to de novo whole-genome sequencing (Fig. 1A**)**. Re-analysis suggests that a His^+^ isolate was used for SHU experiments while a His^−^ isolate was used for MHBG6P, suggesting that the second INF018 freezer stock also contained a mixed population (Fig. 1A and 2). However, *his* operon reads in the MHBG6P 24-h passaged genome were absent in the 48-h passaged genome, indicating a gradual loss in the operon over time (Fig. 2).

### Estimating Auxotrophies from Public Genome Data

**Fig. 3:**
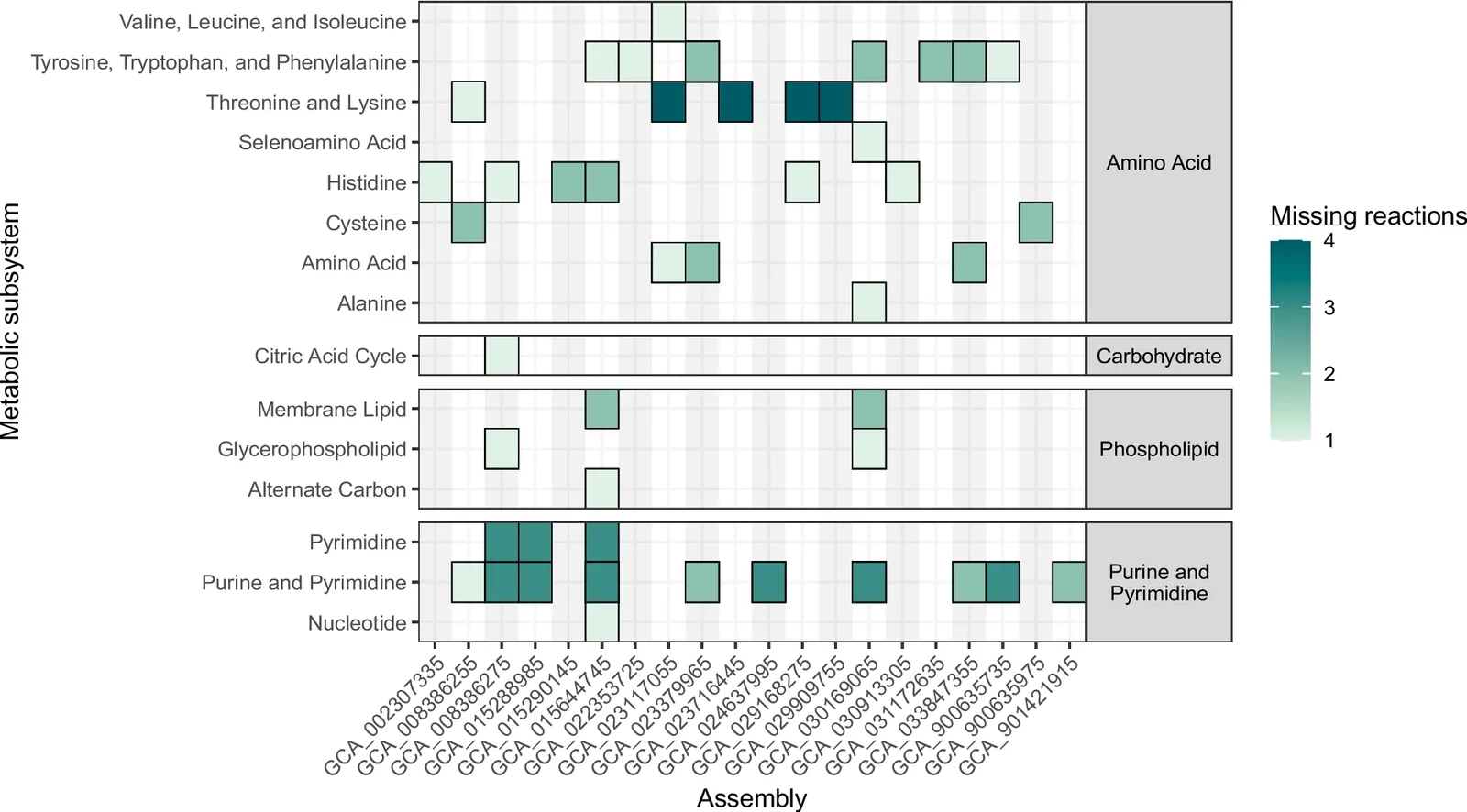
Metabolism auxotrophies identified in the 20 putatively auxotrophic isolates. Counts of individual missing reactions are shown by colour. Auxotrophs are split by the subsystem and general metabolic class. Median missing reactions resulting in substrate auxotrophism was 3 (range 1 to 25)

Given that we had identified two L-histidine auxotrophs among a collection of just 507 isolates (0.4%) [13], we sought to estimate the general prevalence of such auxotrophs among public genome collections. We constructed metabolic models for all complete [21] *Kp*SC genomes in Genbank (*n* = 2637). Twenty (0.8%) were predicted as auxotrophs on M9 + D-glucose media, with the most frequent auxotrophies being purine and pyrimidine biosynthesis and amino acid metabolism (Fig. 3).

## Discussion

Our data highlights how the use of rich culture media can facilitate and conceal genetic mutations that impact bacterial growth capabilities and clinically relevant traits. This has implications for the broad suite of research and diagnostics processes that rely on the use of rich medias, and which may be misled by analysis of mutant strains that do not reflect wild-type characteristics. Example undesirable outcomes may include misrepresentations of ecological roles, molecular epidemiology or antimicrobial susceptibilities, which are known to be influenced by metabolic state [25, 26].

Loss of plasmids during culturing [5, 27, 28] and spontaneous mutants with varying colony morphology [29, 30] have been described previously, as have passage-induced mutations in historical and globally distributed laboratory strains [31, 32]. For example, passage of *Burkholderia pseudomallei* strain K96243 in LB broth for 28 days impacted virulence, protein expression and regulation of metabolism [32]. However, to our knowledge there are few discussions of spontaneous nutrient auxotrophs occurring over the timescale of standard clinical microbiological diagnostic protocols and/or overnight cultures for DNA extractions.

Current recommendations for *Klebsiella* isolation include growth in LB + ampicillin then Simmons Citrate + Inositol [33] or MacConkey/CHROMagar/horse blood agar [34]. The two isolates used in this study (*K. pneumoniae* INF018 and *K. variicola* INF232) were originally collected from patient samples plated onto MacConkey then grown in LB broth prior to sequencing in 2013 [5]. These rich medias would support the within-isolate heterogeneity we observed, eventually resulting in the complete loss of His^+^ sub-populations. However, it is also possible that both sub-populations were present in the patient (urine is histidine rich), but in either case the use of rich media enabled the His^−^ population to persist and outcompete His^+^ populations undetected [12]. We therefore propose that these spontaneous auxotrophies act like *‘*quiet mutations’—a phenotypic parallel to silent mutations. These occur when DNA changes cause a phenotype alteration that remains unnoticed in standard culture conditions.

Our analysis predicted at least 0.8% of completed, high-quality *Kp*SC genomes represent isolates with ≥ 1 auxotrophy (Fig. 3), consistent with data published from a smaller analysis of ~ 230 genomes [35] and indicating that the phenomenon is rare but not insignificant considering the scale of modern genomic sequencing. i.e. 100,000 K*. pneumoniae* genomes are now available in NCBI Pathogens. Similar analyses of four other Gram-negative genera (*Escherichia*, *Pseudomonas*, *Salmonella* and *Yersinia*) predicted between 1.2 and 4.5% were auxotrophic for one or more nutrients [35]. However, it is not possible to determine whether these reflect ‘quiet’ mutations acquired during laboratory culture or otherwise, or genuine biological adaptations. We acknowledge that adaptive loss of biosynthetic pathways has been reported in *E. coli* and other bacterial species (36, 37), especially in specific host-species combinations.

Like the previous study, our investigation of public genomes leveraged reference-based metabolic reconstructions, which may not capture the true extent of metabolic capabilities and associated genetic diversity in the population. As a result, we may have overestimated the prevalence of nutrient auxotrophies. However, we argue that we have likely underestimated the total burden of quiet mutations because our approach focused solely on metabolic gene content, but we expect that non-metabolic gene content would also be impacted. The likelihood of propagation and detection of these mutations is highly dependent on media and growth conditions. Our cautionary tale has served as a good self-reminder when studying microbial ecology to ensure we are using the most appropriate conditions to observe the wild-type organism.

## Data Availability

Reads and assemblies were deposited at Genbank (BioProject PRJNA1115910). Additional supporting code and details found at Figshare (10.6084/m9.figshare.25864864).
